# Comparative analysis of exon 10 and non-exon 10 variants in children with familial mediterranean fever: a retrospective cohort study

**DOI:** 10.1007/s00431-026-06835-4

**Published:** 2026-03-09

**Authors:** Bengisu Menentoğlu, Selen Duygu Arık, Pınar Prencuva Akyürek, Özlem Akgün, Nuray Aktay Ayaz

**Affiliations:** Division of Pediatric Rheumatology, Department of Pediatrics, Istanbul Faculty of Medicine, Istanbul University, Istanbul, Türkiye

**Keywords:** Familial Mediterranean fever, MEFV genotype, Phenotype, Pediatrics

## Abstract

Familial Mediterranean fever (FMF) is the most common inherited autoinflammatory disorder caused by MEFV variants. Exon 10 variants, particularly M694V, are strongly associated with severe disease, but the impact of non–exon 10 variants in children remains less defined. To evaluate genotype–phenotype correlations in pediatric FMF by comparing patients with homozygous exon 10, compound heterozygous exon 10, and exon 10 combined with non–exon 10 variants. This single-center retrospective cohort study included 477 children fulfilling Eurofever/PRINTO criteria between 2018 and 2025. Patients were stratified into four genotype groups. Clinical features, laboratory parameters, and treatments were compared. Disease severity was assessed with the International Severity Scoring System for FMF (ISSF). Homozygous exon 10 variants were associated with an earlier disease onset, shorter diagnostic delay, higher ISSF scores, and a markedly greater requirement for anti–IL-1 therapy compared with other genotypic categories. Patients with compound exon 10 variants and those with combined exon 10–non–exon 10 variants exhibited broadly comparable overall disease severity, although differences were observed in selected clinical manifestations. In contrast, individuals with combined exon 10–non–exon 10 variants demonstrated higher attack frequency, increased inflammatory marker levels, and higher ISSF scores when compared with patients with a single exon 10 allele, supporting a modifier effect of non–exon 10 variants on disease expression.

*Conclusion*: Pediatric FMF patients homozygous for exon 10, especially M694V, show a more severe clinical course with higher disease activity and greater biologic treatment requirements. Exon 10–non–exon 10 variants present with attenuated but clinically relevant phenotypes, resembling compound exon 10 carriers. These findings emphasize the prognostic importance of comprehensive MEFV genotyping in risk stratification and individualized management.

**Table:** 

**What is Known:** • *Familial Mediterranean fever (FMF) is the most common monogenic autoinflammatory disorder, caused by MEFV gene mutations.* • *Exon 10 mutations, particularly M694V homozygosity, are associated with more severe disease, higher attack frequency, colchicine resistance, and increased risk of complications such as amyloidosis.*
**What is New:** • *This is one of the largest pediatric FMF cohorts systematically stratified into homozygous exon 10, compound heterozygous exon 10, exon 10–non–exon 10 groups, and single exon 10 allele* • *Exon 10–non–exon 10 variant combinations were associated with a clinically relevant inflammatory phenotype resembling compound exon 10 genotypes, suggesting a potential modifier role of non–exon 10 variants.*

**Supplementary Information:**

The online version contains supplementary material available at 10.1007/s00431-026-06835-4.

## Introduction

Familial Mediterranean fever (FMF) is the most prevalent monogenic autoinflammatory disease, arising from pathogenic variants in the *MEFV* gene, which is located on the short arm of chromosome 16 and composed of 10 exons [1, 2]. Given its strong genetic background and high prevalence in Mediterranean populations, including Armenians, Arabs, Jews, and Turks, the disease derives its name from its historical roots and original regional predominance, although it is now recognized worldwide [3]. Clinically, FMF is characterized by recurrent, self-limiting episodes of fever, abdominal and chest pain, arthritis, and erysipelas-like erythema [4]. Among the complications is reactive AA amyloidosis, which develops as a consequence of prolonged exposure to elevated serum amyloid A levels, leading to tissue deposition and subsequent organ damage. Colchicine is regarded as the cornerstone of FMF management, as it decreases attack frequency and mitigates the risk of developing secondary amyloidosis, the most severe complication of the disease [5]. Despite its proven efficacy, approximately 5–10% of patients demonstrate resistance or intolerance, for whom anti-IL-1 therapies constitute the recommended alternative [6, 7].

Among the proposed diagnostic tools, the Eurofever/PRINTO classification criteria [8], introduced in 2019, integrate both clinical and genetic features to facilitate the diagnosis of FMF. Pathogenic variants of the *MEFV* gene cluster predominantly in exon 10; however, additional variants in exons 2, 3, and 5 have also been implicated in disease expression [3, 9]. Importantly, accumulating evidence suggests that the type and location of *MEFV* variants not only determine the clinical presentation but also influence disease severity, treatment response, and long-term outcomes. In particular, exon 10 variants, most notably M694V, are strongly associated with severe phenotypes, compound heterozygous exon 10 mutations are linked with variable disease expression, and non–exon 10 variants appear to contribute to heterogeneity, although their impact is less clearly defined [10].

In particular, previous large pediatric FMF cohorts have predominantly focused on single mutations or broad exon-based comparisons, often grouping compound heterozygous and mixed exon genotypes together. Consequently, it remains unclear whether compound exon 10 variants and exon 10–non–exon 10 combinations represent clinically distinct phenotypes or share comparable disease characteristics. Moreover, data on how these genetic subgroups differ in terms of diagnostic delay, disease severity scoring, and biologic treatment requirement in real-life pediatric practice are limited.

Therefore, the present study addresses these gaps by providing a systematic four-group genotypic stratification in one of the largest single-center pediatric FMF cohorts, aiming to delineate clinically meaningful differences beyond previously established associations.

## Materials and methods

### Study design and patients

This single-center retrospective cohort study was conducted between January 2018 and February 2025 at the pediatric rheumatology clinic, where patients were routinely followed at intervals of 3–6 months. A minimum clinical follow-up period of 12 months was required. Pediatric patients with a confirmed diagnosis of FMF according to the Eurofever/PRINTO classification criteria were eligible for inclusion [8]. According to these criteria, the presence of a pathogenic or likely pathogenic *MEFV* variant together with at least one of the following clinical features was required: typical episode duration of 1–3 days, arthritis, chest pain, or abdominal pain. As part of their diagnostic evaluation, all patients underwent *MEFV* gene analysis using next-generation sequencing (NGS). Sequencing targeted all coding exons and exon–intron boundaries of the MEFV gene. Genomic DNA was extracted from peripheral blood samples. NGS achieved a mean sequencing depth exceeding 200 ×, with more than 99% of target regions covered at ≥ 20 ×. Reads were aligned to the GRCh37/hg19 reference genome, and variants were identified and annotated using a standardized bioinformatic pipeline. Patients were stratified into three distinct subgroups according to their *MEFV* genotypic profile. Variant pathogenicity was assessed using the INFEVERS database [11]. Parental genetic analysis confirmed that the two MEFV variants were located on different alleles (in trans), consistent with true compound heterozygosity. The first group consisted of patients with exon 10 homozygous mutations. The second group included patients with biallelic mutations located exclusively in exon 10 in a compound heterozygous state. To explore the potential modifying or low-penetrance role of non–exon 10 variants, Group 3 was defined to include patients carrying an exon 10 mutation together with a non–exon 10 variant not classified as pathogenic or likely pathogenic. In addition, patients who fulfilled the FMF classification criteria and had a single heterozygous MEFV variant located in exon 10 were identified. Although these patients were not included in the primary genotype–phenotype analysis, they were analyzed as a separate group (Group 4) in a secondary comparative analysis to explore the potential clinical and functional relevance of non–exon 10 variants when co-occurring with exon 10 mutations.

### Data collection and variables

For each patient, demographic characteristics such as age, sex, family history of FMF, and parental consanguinity were recorded. Clinical manifestations were documented at the time of diagnosis, and comorbidities were noted if patients had coexisting conditions requiring chronic follow-up. The presence of long-term complications, particularly amyloidosis, was also documented. Disease severity was assessed using the International Severity Scoring System for FMF (ISSF) at the last follow-up visit [12]. Treatment-related data comprised colchicine response, the presence of colchicine resistance, and the need for biologic therapy. Laboratory parameters, including C-reactive protein (CRP) and serum amyloid A (SAA) levels, were retrieved both during attacks and between attacks to evaluate the inflammatory burden and disease activity over time. For reference, a CRP level above 5 mg/L and a serum amyloid A level above 6.4 mg/L were considered elevated.

### Statistical analysis

Data were analyzed using the Statistical Package for the Social Sciences (SPSS) version 26.0 (IBM Corp., Armonk, NY, USA). The distribution of numerical variables was investigated through both visual means (histograms and probability plots) and analytical methods, such as the Kolmogorov–Smirnov/Shapiro–Wilk tests. Descriptive statistics for numerical variables included the minimum, maximum, mean, standard deviation, interquartile ranges (IQR), and median values, reported according to their respective distributions. Categorical variables were analyzed using frequency and percentage distributions. The Chi-square test or Fisher's exact test, with Bonferroni correction for multiple comparisons, was applied to compare categorical variables. Quantitative variables were compared using the Kruskal–Wallis test and the Mann–Whitney U test, depending on their distribution. A p-value below 0.05 was considered statistically significant. Multivariable logistic regression analyses were performed to identify factors associated with anti–IL-1 use and high disease severity (ISSF ≥ 3). The models were adjusted for age at disease onset and follow-up duration. Results were reported as odds ratios (ORs) with 95% confidence intervals (CIs). Missing data were < 5% for all variables, and complete-case analysis was performed.

### Ethical approval

Written informed consent was obtained from the parents or legal guardians of all participants in accordance with the Declaration of Helsinki; this consent covered genetic testing and the use of anonymized genetic and clinical data for research purposes. The study protocol was approved by the Ethics Committee of Istanbul University (approval number: 3429662).

## Results

### Demographic and clinical features

A total of 477 patients were included, consisting of 232 females (48.6%) and 245 males (51.4%). The median age at disease onset was 48 (IQR 24–72) months, and the median age at diagnosis was 63 (IQR 40–96) months. The median follow-up duration was 96 (IQR 72–120) months. A family history of FMF was present in 321 patients (67.3%), and parental consanguinity was reported in 120 patients (25.2%). Comorbid conditions were identified in 38 patients (8%). The most frequent was juvenile idiopathic arthritis, observed in 15 patients (3.1% of the cohort). Other comorbidities included asthma (*n* = 6), IgA vasculitis (*n* = 4), hypothyroidism (*n* = 3), epilepsy (*n* = 3), inflammatory bowel disease (*n* = 2), migraine (*n* = 2), autism spectrum disorder (*n* = 2), and Addison’s disease (*n* = 1).

The most common clinical manifestations were fever (87%) and abdominal pain (82.4%), followed by arthritis (24.5%), myalgia (24.9%), chest pain (24.3%), and erysipelas-like erythema (13.6%). Patients experienced a median of 12 (IQR 6–18) attacks per year, with a median duration of 2 (IQR 2–3) days, and the median ISSF score at the last visit was 2 (IQR 1–3). The median levels of CRP and SAA during attacks were 52 (IQR 34–102) mg/L and 52.5 (IQR 22–144) mg/L, respectively. In the attack-free period, median CRP and SAA levels were 1.2 (IQR 0.6–2.9) mg/L and 1.6 (IQR 0.6–3.5) mg/L, respectively.

At the time of analysis, 431 patients (90.1%) were receiving colchicine, 55 patients (11.5%) were on combined colchicine and anti-IL-1 therapy, and one patient was treated with colchicine and tocilizumab due to concomitant juvenile idiopathic arthritis. Colchicine-related adverse effects were documented in 20 patients (4.2%) across the cohort. Gastrointestinal symptoms represented the most frequent colchicine-related side effect, observed in 15 patients (3.1%), followed by elevations in liver function tests in 7 patients (1.5%). A summary of the demographic, clinical, and laboratory characteristics of the entire cohort is presented in Table 1.

**Table 1:** Demographic, clinical, and laboratory characteristics of the study cohort

Demographic features	
Gender	
Female n, (%)	232 (48.6)
Male n, (%)	245 (51.4)
Age at onset (month) (median) (IQR 25–75)	48 (24–72)
Age at diagnosis (month) (median) (IQR 25–75)	63 (40–96)
Time to diagnosis (month) (median) (IQR 25–75)	12 (6–24)
Duration of disease follow-up (month) (median) (IQR 25–75)	96 (72–120)
Family history of FMF n, (%)	321 (67.3)
Parental consanguinity n, (%)	120 (25.2)
**Characteristics of attacks**	
Fever n, (%)	415 (87)
Abdominal pain n, (%)	393 (82.4)
Chest pain n, (%)	116 (24.3)
Arthritis n, (%)	117 (24.5)
Erysipelas like erythema n, (%)	65 (13.6)
Pleural effusion n, (%)	3 (0.8)
Exercise induce leg pain n, (%)	68 (14.3)
Myalgia n, (%)	119 (24.9)
Protracted febrile myalgia n, (%)	2 (0.5)
Orchitis n, (%)	6 (1.3)
Headache n, (%)	17 (3.6)
**Disease severity, laboratory findings, and treatment**	
Duration of attacks (day) (median) (IQR 25–75)	2 (2–3)
Number of attacks per year (median) (IQR 25–75)	12 (6–18)
CRP mg/L during attacks (median) (IQR 25–75)	52 (34–102)
SAA mg/L during attacks (median) (IQR 25–75)	52.5 (22–144)
CRP mg/L between attacks (median) (IQR 25–75)	1.2 (0.6–2.9)
SAA mg/L between attacks (median) (IQR 25–75)	1.6 (0.6–3.5)
ISSF score (median) (IQR 25–75)	2 (1–3)
Anti-IL1 treatment n, (%)	55 (11.5)

*Anti-IL-1* Anti–interleukin-1 therapy, *CRP* C-reactive protein, *FMF* familial Mediterranean fever, *IQR* interquartile range, *ISSF* International Severity Scoring System for FMF, *SAA* Serum amyloid A

### MEFV gene variants of the study population

**Fig. 1:**
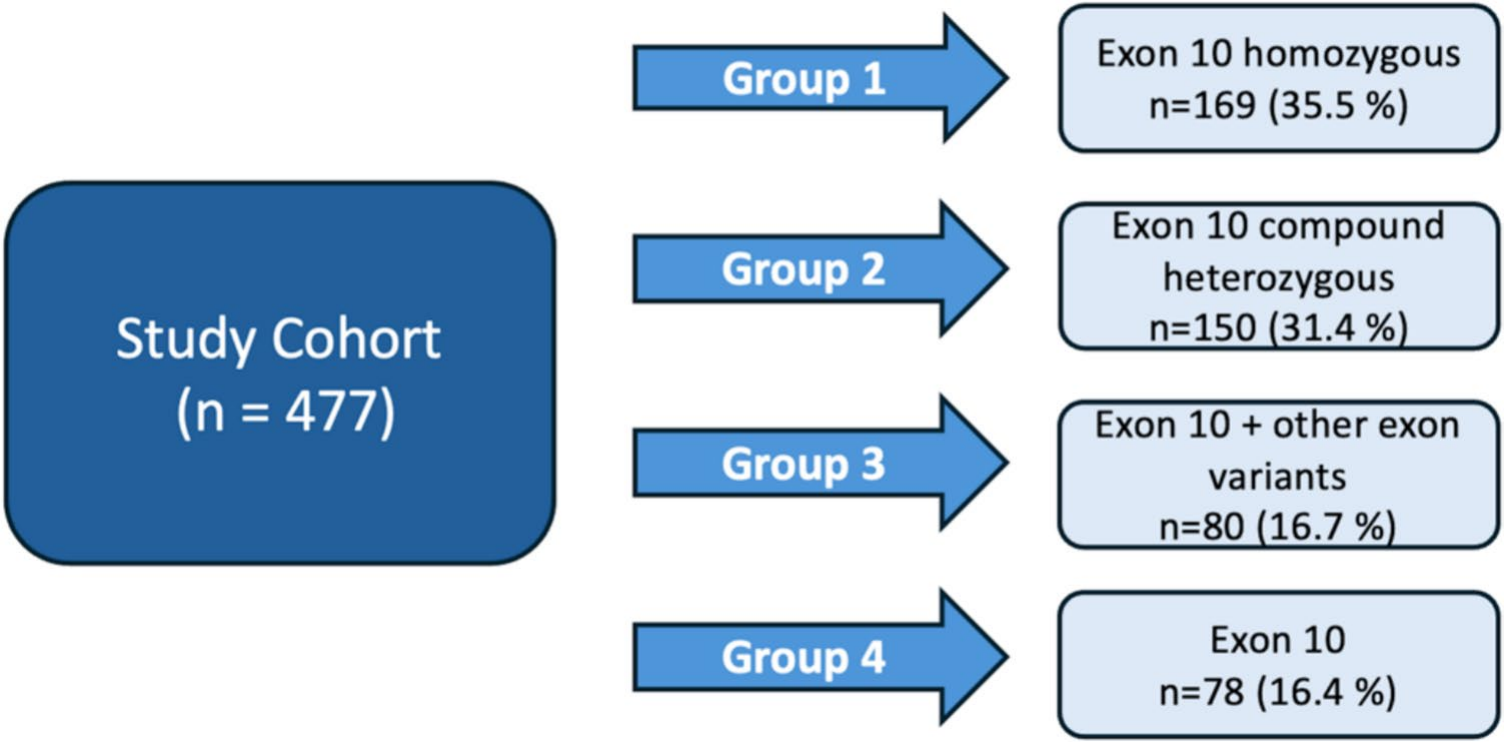
Schematic representation of genotypic stratification into three patient groups

In our cohort, the distribution of MEFV variants was as follows: 169 patients (35.5%) had homozygous variants in exon 10, 150 patients (31.4%) had compound heterozygous variants in exon 10, 80 patients (16.7%) had combined exon 10 and non–exon 10 variants, and 78 patients (16.4%) had a single exon 10 allele (Fig. 1). The most common genotype was homozygous M694V (*n* = 146, 30.6%), followed by compound heterozygous M694V–M680I (*n* = 59, 12.4%), a single exon 10 M694V allele (*n* = 48, 10.1%), and M694V–R202Q (*n* = 46, 9.6%). The detailed distribution of variants within each genotypic group is provided in the Supplementary Table.

### Clinical and demographic comparisons by genotype

When clinical and demographic variables were compared across Groups 1–3, no significant difference was observed in sex distribution (*p* = 0.24). Group 1 showed a trend toward earlier age at symptom onset compared to the other groups, although this did not reach statistical significance (*p* = 0.052). Age at diagnosis was similar across all three groups (*p* = 0.130). The median duration of follow-up was significantly longer in Group 1 (90 months) compared to Group 2 (69 months) and Group 3 (66.5 months) (*p* = 0.015). The median time to diagnosis was 10 months in Group 1, 10.5 months in Group 2, and 16 months in Group 3, indicating significantly shorter times in Groups 1 and 2 compared to Group 3 (*p* < 0.001). Parental consanguinity was significantly more frequent in Group 1 (34.3%) compared to Group 2 (18.7%) and Group 3 (20.0%) (*p* = 0.030), while the presence of a family history of FMF did not differ significantly among groups (*p* = 0.586).

Abdominal pain was reported in 84% of Group 1, 89.2% of Group 2, and 71.3% of Group 3, with significantly higher frequencies in Groups 1 and 2 compared to Group 3 (*p* = 0.002 for both). Chest pain was observed in 32.0% of Group 1, 22.7 0% of Group 2, and 13.8% of Group 3, with a significantly higher rate in Group 1 compared to Group 3 (*p* = 0.006), while no other pairwise differences were significant. Arthritis was present in 39.1% of Group 1, 20.0% of Group 2, and 17.5% of Group 3, being significantly more frequent in Group 1 compared to the other groups (*p* < 0.001). Similarly, erysipelas-like erythema was observed in 25.9%, 8.7%, and 3.8% of the respective groups, with significantly higher frequency in Group 1 (*p* < 0.001). Exercise-induced leg pain occurred in 23.4% of Group 1, 15.3% of Group 2, and 3.8% of Group 3, with significantly higher frequencies in Group 1 and Group 2 compared to Group 3 (*p* < 0.001 and *p* = 0.008, respectively). Renal amyloidosis was detected in one patient in Group 1 (M694V homozygous), while splenomegaly was identified in three patients overall, including one in Group 1 (M694V/M694V) and two in Group 2 (M694V/M680I and M694V/V726A). The median ISSF score was 4 (IQR: 2–4) in Group 1, 2 (IQR: 1–3) in Group 2, and 2 (IQR: 1–2) in Group 3, with a significantly higher score in Group 1 compared to the other groups (*p* < 0.001, r = 0.31). Similarly, the proportion of patients receiving anti-IL-1 therapy was 24.3% in Group 1, 6.7% in Group 2, and 5.0% in Group 3, being significantly higher in Group 1 than in Groups 2 and 3 (*p* < 0.001, Cramér’s V = 0.26) (Table 2).

**Table 2:** Comparison of clinical features of patients according to *MEFV* genotype

	Group 1 (*n* = 169)	Group 2 (*n* = 150)	Group 3 (*n* = 80)	*p*
Demographic features				
Gender				
Female n, (%)	85 (50.3)	76 (50.7)	32 (40)	0.24
Male n, (%)	84 (49.7)	74 (49.3)	48 (60)	
Age at onset (month) (median) (IQR 25–75)	40 (20.5–69)	50 (29.7–74.7)	48 (12–82)	0.052
Age at diagnosis (month) (median) (IQR 25–75)	60 (36–90)	64 (44–93)	67.5 (42–97.5)	0.13
Time to diagnosis (month) (median) (IQR 25–75)	10 (6–22.5)	10.5 (6–20)	16 (10–24)	** < *0.001***
Duration of disease follow-up (month) (median) (IQR 25–75)	90 (45–125)	69 (35.5–111)	66.5 (36–99)	***0.015***
Family history of FMF n, (%)	117 (69.2)	99 (66)	58 (72.5)	0.58
Parental consanguinity n, (%)	58 (34.3)	28 (18.7)	16 (20)	***0.03***
Characteristics of attacks				
Fever n, (%)	150 (88.8)	137 (91.3)	68 (85)	0.34
Abdominal pain n, (%)	142 (84)	134 (89.2)	57 (71.3)	***0.02***
Chest pain n, (%)	54 (32)	34 (22.7)	11 (13.8)	0.06
Arthritis n, (%)	66 (39.1)	30 (20)	14 (17.5)	** < *0.001***
Erysipelas-like erythema n, (%)	43 (25.9)	13 (8.7)	3 (3.8)	** < *0.001***
Exercise induce leg pain n, (%)	39 (23.4)	23 (15.3)	3 (3.8)	** < *0.001***
Myalgia n, (%)	49 (29.3)	39 (26)	23 (23.8)	0.79
Orchitis n, (%)	2 (1.2)	3 (2)	1 (1.3)	0.87
Headache n, (%)	5 (3)	5 (3.3)	5 (6.3)	0.42
Disease severity, laboratory findings, and treatment				
Duration of attacks (day) (median) (IQR 25–75)	2 (2–3)	2 (2–3)	2 (2–3)	0.08
Number of attacks per year (median) (IQR 25–75)	12 (6–13.5)	12 (8–15)	12 (10–12)	0.9
CRP mg/L during attacks (median) (IQR 25–75)	63 (43.2–103.7)	67 (35.5–102.5)	56 (42.7–84.5)	0.39
SAA mg/L during attacks (median) (IQR 25–75)	85 (28.9–201.2)	43.8 (14.6–185)	82 (24.4–128.5)	0.34
CRP mg/L between attacks (median) (IQR 25–75)	1.7 (0.6–3.4)	1.1 (0.6–2.3)	0.9 (0.4–2.1)	0.06
SAA mg/L between attacks (median) (IQR 25–75)	2.3 (0.5–4.5)	2.4 (0.9–3.5)	2.8 (0.6–3.5)	0.86
ISSF score (median) (IQR 25–75)	4 (2–4)	2 (1–3)	2 (1–2)	** < *0.001***
Side effect of colchicine n, (%)	9 (5.4)	8 (5.4)	0 (0)	0.1
Anti-IL1 treatment n, (%)	41 (24.3)	10 (6.7)	4 (5)	** < *0.001***

*Anti-IL-1* Anti–interleukin-1 therapy, *CRP* C-reactive protein, *FMF* familial Mediterranean fever, *IQR* interquartile range, *ISSF* International Severity Scoring System for FMF, *SAA* serum amyloid A,

Statistically significant p values are shown in bold

An additional comparison was performed between Group 3 and a separately defined Group 4, consisting of patients with a single exon 10 allele, to explore whether the presence of a single exon 10 variant alone could account for this pattern.

When Group 3 and Group 4 were compared, no significant differences were observed in sex distribution (*p* = 0.64), family history of FMF (*p* = 0.103), or parental consanguinity (*p* = 0.638). Patients in Group 4 had a significantly later age at symptom onset compared to Group 3, with a median age at onset of 56 (IQR 28–94.5) months versus 47 (IQR 12.5–71) months (*p* = 0.043), while age at diagnosis and time to diagnosis were similar between the groups (*p* = 0.314 and *p* = 0.102, respectively). The duration of disease follow-up was significantly shorter in Group 4, with a median of 48 (IQR 32–71.3) months, compared to 66.5 (IQR 36–99) months in Group 3 (*p* = 0.016).

Regarding attack characteristics, chest pain, erysipelas-like erythema, and myalgia were significantly more frequent in Group 3 than in Group 4 (p = 0.006, p = 0.006, and p = 0.003, respectively), whereas the frequencies of fever, abdominal pain, arthritis, exercise-induced leg pain, orchitis, and headache did not differ significantly between the groups.

The number of attacks per year was significantly greater in Group 3 compared to Group 4, with a median of 12 (IQR 10–12) attacks per year versus 8 (IQR 6–12) attacks per year (*p* < 0.001). During attacks, median CRP and SAA levels were significantly higher in Group 3 (p < 0.001 for both), and SAA levels between attacks also remained higher in Group 3 (*p* < 0.001), while median CRP levels between attacks were comparable between the groups (p = 0.986). In parallel, the median ISSF score was higher in Group 3, with a median of 2 (IQR 1–2), compared to 1 (IQR 1–2) in Group 4 (*p* = 0.005) (Table 3).

**Table 3:** Comparison of clinical and laboratory characteristics between Group 3 and Group 4

	Group 3 (*n* = 80)	Group 4 (*n* = 78)	*p*
Demographic features			
Gender			
Female n, (%)	37 (46.3%)	39 (50.0%)	
Male n, (%)	43 (53.7%)	39 (50.0%)	0.64
Age at onset (month) (median) (IQR 25–75)	47 (12.5–71)	56 (28–94.5)	***0.043***
Age at diagnosis (month) (median) (IQR 25–75)	65 (43.5–94)	76.5 (46–109.3)	0.314
Time to diagnosis (month) (median) (IQR 25–75)	16 (10–24)	14 (9–18.5)	0.102
Duration of disease follow-up (month) (median) (IQR 25–75)	66.5 (36–99)	48 (32–71.3)	***0.016***
Family history of FMF n, (%)	58 (72.5%)	47 (60.3%)	0.103
Parental consanguinity n, (%)	16 (20.0%)	18 (23.1%)	0.638
Characteristics of attacks			
Fever n, (%)	68 (85.0%)	60 (76.9%)	0.18
Abdominal pain n, (%)	57 (71.3%)	60 (76.9%)	0.416
Chest pain n, (%)	34 (42.5)	17 (21.8)	***0.006***
Arthritis n, (%)	14 (17.5)	7 (9.0)	0.115
Erysipelas-like erythema n, (%)	19 (23.8)	6 (7.7)	***0.006***
Exercise induce leg pain n, (%)	3 (3.8%)	3 (3.8%)	0.975
Myalgia n, (%)	23 (28.8)	8 (10.3)	***0.003***
Orchitis n, (%)	1 (1.3)	0 (0)	-
Headache n, (%)	5 (6.3%)	2 (2.6%)	0.260
Disease severity, laboratory findings, and treatment			
Duration of attacks (day) (median) (IQR 25–75)	2 (2–3)	2 (1–3)	***0.042***
Number of attacks per year (median) (IQR 25–75)	12 (10–12)	8 (6–12)	** < *0.001***
CRP mg/L during attacks (median) (IQR 25–75)	56 (42.8–84.5)	33 (25–45)	** < *0.001***
SAA mg/L during attacks (median) (IQR 25–75)	82 (24.4–128.5)	0 (0–16.3)	** < *0.001***
CRP mg/L between attacks (median) (IQR 25–75)	0.92 (0.48–2.10)	0.80 (0.50–2.85)	0.986
SAA mg/L between attacks (median) (IQR 25–75)	2.8 (0.6–3.6)	0.7 (0–2.4)	** < *0.001***
ISSF score (median) (IQR 25–75)	2 (1–2)	1 (1–2)	***0.005***
Side effect of colchicine n, (%)	3 (3.9)	1 (1.3)	0.62
Anti-IL1 treatment n, (%)	4 (5.0%)	0 (0%)	0.12

*Anti-IL-1* Anti–interleukin-1 therapy, *CRP* C-reactive protein, *FMF* familial Mediterranean fever, *IQR* interquartile range, *ISSF* International Severity Scoring System for FMF, *SAA* serum amyloid A

Statistically significant p values are shown in bold

Multivariable logistic regression analyses were performed to determine whether the observed genotype–phenotype associations were independent of follow-up duration and age at disease onset. After adjustment, the homozygous exon 10 genotype remained a strong independent predictor of both high disease severity (ISSF ≥ 3) and anti–IL-1 requirement. Specifically, patients with homozygous exon 10 variants had 4.41-fold increased odds of high ISSF scores (OR 4.41, 95% CI 2.90–6.69, *p* < 0.001) and 5.23-fold increased odds of anti–IL-1 therapy use (OR 5.23, 95% CI 2.65–10.30, *p* < 0.001) compared with patients carrying other genotypes. Longer follow-up duration was independently associated with both outcomes (ISSF ≥ 3: OR 1.01 per month increase, 95% CI 1.005–1.014, *p* < 0.001; anti–IL-1 use: OR 1.02 per month increase, 95% CI 1.01–1.03, *p* < 0.001), whereas age at disease onset was not a significant predictor in either model (Table 4).

**Table 4:** Independent association of homozygous exon 10 genotype with high disease severity (ISSF ≥ 3) and Anti–IL-1 requirement in multivariable logistic regression analysis

Predictor	High ISSF (≥ 3) OR (95% CI)	*p* value	Anti–IL-1 Use OR (95% CI)	*p* value
Homozygous exon 10 genotype	4.41 (2.90–6.69)	< 0.001	5.23 (2.65–10.30)	< 0.001
Follow-up duration (per month)	1.01 (1.005–1.014)	< 0.001	1.02 (1.01–1.03)	< 0.001
Age at disease onset (per month)	0.997 (0.991–1.003)	0.353	0.991 (0.980–1.002)	0.101

*OR* Odds ratio, *CI* Confidence interval, *ISSF* International Severity Scoring System for Familial Mediterranean Fever

Models adjusted for follow-up duration and age at disease onset

## Discussion

Consistent with previous pediatric FMF studies, our analysis highlights the prominent impact of exon 10 mutations particularly M694V homozygosity on disease expression. Children with homozygous exon 10 variants demonstrated a more severe clinical profile, reflected by higher ISSF scores and a greater need for anti-IL-1 therapy compared with those harboring compound heterozygous exon 10 variants or exon 10 combined with non–exon 10 mutations. This aligns with earlier reports indicating that M694V homozygosity is associated with increased disease activity and colchicine resistance [1, 10, 13, 14].

The multivariable analyses further confirmed that homozygous exon 10 genotype is an independent determinant of disease severity in pediatric FMF. Even after adjustment for follow-up duration and age at disease onset, exon 10 homozygosity remained strongly associated with both higher ISSF scores and increased requirement for anti–IL-1 therapy. These findings indicate that the increased disease burden observed in this subgroup reflects a true genotype-driven effect rather than differences in clinical observation time, reinforcing the prognostic value of comprehensive MEFV genotyping in risk stratification.

Arthritis and erysipelas-like erythema were significantly more common in Group 1, whereas these manifestations were less frequent in the other subgroups. Previous studies have yielded comparable results, suggesting an association between M694V homozygosity and articular as well as cutaneous involvement [15–17]. In our cohort, abdominal pain was relatively less frequent among patients in Group 3, suggesting a more heterogeneous and occasionally attenuated phenotype in this subset. Within our dataset, age at disease onset tended to be younger in Group 1, although this difference did not reach statistical significance. By contrast, the diagnostic delay was notably shorter in patients in Group 1 and Group 2 compared with those in Group 3. This pattern may reflect a more classical and readily recognizable clinical presentation associated with exon 10–related genotypes, whereas genotypes involving non–exon 10 variants may lead to diagnostic delays, as similarly reported in other pediatric studies [18, 19]. Parental consanguinity was significantly more common in Group 1, consistent with the autosomal recessive inheritance of FMF and the influence of consanguinity on its prevalence in endemic regions. The frequency of a positive family history was comparable across all groups, indicating that while FMF frequently clusters within families, the presence of a family history does not appear to be determined by specific genotypes.

Inflammatory markers were broadly similar between groups. CRP and serum amyloid A levels during and between attacks did not vary significantly, consistent with previous findings that these biomarkers reflect inflammatory activity rather than underlying genotype [20]. Only one patient in the cohort developed amyloidosis, and this case was confined to the homozygous M694V group. The relatively low prevalence of amyloidosis observed in our study is more plausibly attributable to improvements in disease management, including earlier diagnosis, systematic monitoring, and prompt initiation of effective therapies, rather than a true reduction in the underlying risk.

In this comparative analysis, patients in Group 2 and Group 3 demonstrated broadly comparable clinical features. Rates of chest pain, arthritis, and erysipelas-like erythema, as well as ISSF scores and the need for anti–IL-1 therapy, did not differ meaningfully between these two groups, while all of these parameters were lower than those observed in Group 1. Although the overall clinical profile of Group 3 was largely similar to that of Group 2, differences emerged in selected clinical manifestations. Specifically, Group 3 showed lower frequencies of abdominal pain and exercise-induced leg pain. These findings suggest that, while Group 3 does not exhibit a uniformly milder disease course compared with Group 2, certain non–exon 10 variants may influence the expression of specific inflammatory symptoms rather than overall disease severity. This pattern supports the concept that such variants may act as genetic modifiers rather than exerting a uniform pathogenic or neutral effect.

To further clarify whether the observed similarity between Group 2 and Group 3 was driven primarily by the presence of an exon 10 mutation or modified by the accompanying variant, an additional comparison was performed between patients in Group 3 and those carrying a single exon 10 allele (Group 4). This secondary analysis demonstrated that patients in Group 3 exhibited a more inflammatory phenotype than those in Group 4, characterized by higher attack frequency, elevated CRP and SAA levels during and between attacks, and higher ISSF scores. In addition, chest pain, erysipelas-like erythema, and myalgia were significantly more frequent in Group 3, whereas abdominal pain and arthritis did not differ significantly between the two groups. These findings suggest that the presence of an additional non–exon 10 variant may contribute to disease expression when co-occurring with an exon 10 mutation, supporting a modifier rather than a neutral role for such variants. While overall disease severity in Group 3 remained lower than that observed in homozygous exon 10 patients, the comparison with single exon 10 allele carriers highlights that exon 10–non–exon 10 combinations are associated with a clinically more active phenotype than exon 10 heterozygosity alone.

The pathogenic role of some non–exon 10 variants remain controversial. Although most non–exon 10 MEFV variants are generally associated with milder phenotypes, certain variants located outside exon 10, such as H478Y in exon 5 and T577A in exon 8, have been reported to be associated with severe inflammatory disease courses, underscoring that non–exon 10 variants are not uniformly benign [21, 22]. Moradian et al. emphasized that the location of MEFV mutations, particularly in exon 10, rather than the substitution type, is the key determinant of disease severity, implying that non–exon 10 variants may act as modifiers when combined with exon 10 mutations [23]. Supporting this concept, Endo et al. demonstrated in a Japanese cohort that the coexistence of exon 2 variants with exon 10 mutations was associated with earlier disease onset, increased attack frequency, and elevated IL-18 levels even during remission, highlighting their potential to increase inflammatory burden [24]. Similarly, Awaad et al. showed that the E148Q variant, though usually linked to mild to moderate disease, remains clinically relevant and may contribute to phenotype expression when occurring with exon 10 mutations [25]. Consistent with these observations, Baggio et al. functionally analyzed the R202Q variant and reported that while inflammasome activation did not differ from healthy controls, cytological abnormalities and low-grade inflammation were present, suggesting that this variant may act as a disease modifier rather than a benign polymorphism [26]. Taken together, these studies reinforce that exon 10 non–exon 10 combinations, particularly those involving E148Q or R202Q, can contribute to clinically significant disease expression and should not be disregarded in clinical assessment. When considered together with our findings, these reports suggest that exon 10–non–exon 10 variant combinations may contribute to phenotypes that in some respects resemble those observed in patients with compound exon 10 variants.

Taken together, our findings suggest a graded genotype–phenotype spectrum in pediatric FMF: homozygous exon 10 mutations confer the highest disease severity; compound exon 10 and exon 10–non–exon 10 combinations result in intermediate phenotypes with broadly comparable global severity; and a single exon 10 allele is associated with a milder inflammatory profile. Within this framework, non–exon 10 variants appear to modulate specific clinical features and inflammatory burden rather than uniformly altering disease severity.

This study has several limitations that should be acknowledged. First, it was conducted in a single tertiary center, which may limit the generalizability of the findings to populations with different ethnic and geographic distributions of MEFV variants. The retrospective design precluded evaluation of long-term outcomes beyond the study period. Finally, environmental and epigenetic factors, which may also influence disease expression, were not systematically assessed. Despite these limitations, the large sample size, systematic genetic stratification, and detailed clinical characterization strengthen the validity of our findings.

To our knowledge, this is one of the largest comparative pediatric FMF cohorts analyzed to date, stratified into four genotype groups for systematic evaluation of genotype–phenotype associations. Our findings show that patients homozygous for exon 10 variants, particularly M694V, had the most severe course with greater need for biologic therapy, while non–exon 10 variants in combination with exon 10 mutations also contributed to clinically relevant manifestations. These results underscore the importance of considering the broader mutational context in prognostic evaluation and suggest that integrating mechanistic insights with clinical data may facilitate more precise risk stratification and individualized management in pediatric FMF.

## Supplementary Information

Below is the link to the electronic supplementary material.

Supplementary file1 (DOCX 19 KB)

## Data Availability

The datasets generated during and/or analyzed during the current study are available from the corresponding author on reasonable request.

## Abbreviations

FMF: Familial Mediterranean fever
ISSF: International Severity Scoring System for FMF ISSF
NGS: Next-generation sequencing
CRP: C-reactive protein
SAA: Serum amyloid A
IQR: Interquartile range
Anti-IL-1: Anti–interleukin-1 therapy
