# Supplementary material for: Comparative analysis of exon 10 and non-exon 10 variants in children with familial mediterranean fever: a retrospective cohort study
Source: Eur J Pediatr. 2026 Mar 9;185(3):169. doi: 10.1007/s00431-026-06835-4 (PMC12971933; doi:10.1007/s00431-026-06835-4)
Supplement: Supplementary file 1 — Supplementary file1 (DOCX 19 KB) [file 431_2026_6835_MOESM1_ESM.docx]

**Supplementary Table. Genotypic classification of the study cohort according to *MEFV* variants**

|  | *MEFV* variants | n, (%) |
| --- | --- | --- |
| Group 1 | M694V/M694V  M680I/M680I  V726A/V726A  R761H/R761H  A744S/A744S  M694I/M694I | 146 (86.4)  15 (8.9)  5 (3)  1 (0.6)  1 (0.6)  1 (0.6) |
| Group 2 | M694V/M680I  M694V/V726A  M680I/V726A  M694V/R761H  M680I/R761H  V726A/R761H  M694V/M694I  M694I/M680I  M694V/K695R  M680I/A744S  K695R/V726A | 59 (39.3)  38 (25.3)  21 (14)  15 (10)  5 (3.3)  4 (2.7)  3 (2)  1 (0.6)  2 (1.3)  1 (0.6)  1 (0.6) |
| Group 3 | M694V/R202Q  M694V/E148Q  M680I/E148Q  M694V/P369S  V726A/R202Q  V726A/F491I  K695R/E148Q  M680I/R202Q  M694V/E230K  M694V/I591T  R761H/R202Q  V726A/E148Q | 46 (57.5)  15 (18.8)  4 (5)  3 (3.8)  3 (3.8)  2 (2.5)  1 (1.3)  2 (2.5)  1 (1.3)  1 (1.3)  1 (1.3)  1 (1.3) |
| Group 4 | M694V/-  M680I/-  V726A/- | 48 (61.5)  23 (29.5)  7 (9) |

*Percentages are calculated within each genotype group and indicate the proportion of patients with the specified MEFV variant combination relative to the total number of patients in that group.*
